# Exosomes Regulate the Transformation of Cancer Cells in Cancer Stem Cell Homeostasis

**DOI:** 10.1155/2018/4837370

**Published:** 2018-09-23

**Authors:** Jiasheng Xu, Kaili Liao, Weimin Zhou

**Affiliations:** ^1^Department of Vascular Surgery, The Second Affiliated Hospital of Nanchang University, No. 1 Minde Road, Nanchang 330006, Jiangxi Province, China; ^2^Department of Clinical Laboratory, The Second Affiliated Hospital of Nanchang University, Minde Road, Nanchang, 330006 Jiangxi Province, China

## Abstract

In different biological model systems, exosomes are considered mediators of cell-cell communication between different cell populations. Exosomes, as extracellular vesicles, participate in physiological and pathological processes by transmitting signaling molecules such as proteins, nucleic acids, and lipids. The tumor's microenvironment consists of many types of cells, including cancer stem cells and mesenchymal cells. It is well known that these cells communicate with each other and thereby regulate the progression of the tumor. Recent studies have provided evidence that exosomes mediate the interactions between different types of cells in the tumor microenvironment, providing further insight into how these cells interact through exosome signaling. Cancer stem cells are a small kind of heterogeneous cells that existed in tumor tissues or cancer cell lines. These cells possess a stemness phenotype with a self-renewal ability and multipotential differentiation which was considered the reason for the failure of conventional cancer therapies and tumor recurrence. However, a highly dynamic equilibrium was found between cancer stem cells and cancer cells, and this indicates that cancer stem cells are no more special target and blocking the transformation of cancer stem cells and cancer cells seem to be a more significant therapy strategy. Whether exosomes, as an information transforming carrier between cells, regulated cancer cell transformation in cancer stem cell dynamic equilibrium and targeting exosome signaling attenuated the formation of cancer stem cells and finally cure cancers is worthy of further study.

## 1. Introduction

Exosomes are vesicles of 30 to 100 nm in size originating in the endosomes. Almost all cells release exosomes or extracellular vesicles (EVs) and are present in all body fluids. Exosomes serve as carriers for the exchange of substances between cells, mediate cell-cell communication, and participate in various physiological and pathological processes of the body. Heterogeneity is an important feature of malignant tumors. Cancer stem cells (CSCs) are a subpopulation of tumor cells with self-renewal and differentiation potential. The presence of CSCs leads to failure of traditional treatment and tumor recurrence. However, CSCs are not stable, stationary solid cell populations. Under a certain microenvironment, some differentiated noncancer stem cells (non-CSCs) can regain stemness through dedifferentiation or reprogramming. For phenotypes, CSCs and non-CSCs are in a dynamic equilibrium state of differentiation and dedifferentiation [1]. Cell communication and material exchange between CSCs and other cells in tumor cells and their tumor microenvironment are essential to maintain their homeostasis. Exosomes, as carriers, play an important role in mediating cellular communication and substance exchange between tumor cells and other cells in their tumor microenvironment; they regulate tumor growth, metastasis, drug resistance (by transporting tumor-associated mRNAs, miRNAs, and proteins), angiogenesis, immune escape, and other processes. As an information carrier, exosomes are involved in the transformation between non-CSCs and CSCs and the maintenance of tumor stem cell homeostasis and their mechanisms of action. Whether exosomes can target exosomes and their signaling pathways to eliminate cancer stem cells can be studied further. To this end, the paper discusses the processes of biogenesis and its contents, tumor stem cells, tumor stem cell dynamic balance and its influencing factors, the role of exosomes in maintaining the phenotype of cancer stem cells, and the treatment of exosomes and tumors. A brief review of the research progress is offered to provide a reference for relevant research.

## 2. Exosome Biogenesis

### 2.1. Occurrence and Content Sorting of Exosomes

The term exosome was first proposed by Trams et al. [2] in the early 1980s. The two types of vesicles with diameters of 40 nm and 500–1000 nm that have 5′-nucleotidase activity observed by electron microscopy are called exosomes, and it was suggested that these vesicles may have physiological functions. Subsequent studies revealed the endosomal origin of exosomes [3], and these exosomes were able to carry a variety of signaling molecules [4–6]. The results suggest that exosomes may be important mediators in cell-to-cell communication. With the continuous deepening of their research, a preliminary understanding of the biological processes of exosomes has taken place.

Exosomal biogenesis is a closely ordered process that involves a variety of cellular regulatory mechanisms. First, the cells internalize extracellular ligands or cellular components by endocytosis to form early endosomes. During early maturation, the endosomes form inward luminal vesicles (ILVs) by inward budding. The process of selectively encapsulating proteins, nucleic acids, lipids, etc., transforms early endosomes into multivesicular bodies (MVBs) [7]. A part of the formed multivesicular body is fused with lysosomes and degraded, providing cells with energy substances and structural molecules; the other part is released to the extracellular environment via Golgi recycling or secretion by cells [5].

Exosome contents can be selectively sorted into ILVs by a variety of mechanisms. The endoprotein sorting and transferring device (ESCRT) selectively cleaves ubiquitinated proteins into ILVs. This process requires the participation of ESCRT-0, -I, -II, and -III and VPS4, VTA1, ALIX/PDCD6IP, and TSG101 [8]. Lipids such as ceramide, cholesterol, and the four-transmembrane protein superfamily, like CD9, CD63, and CD81, were also found to mediate exosome protein sorting [9]. Sorting of exosomal mRNAs may be mediated by the Z-zipper structure of its 3′-UTR, whereas miRNAs may be sorted into MVBs through complexes with RISCs [10, 11].

### 2.2. Release and Uptake of Exosomes

After the formation of MVBs, they will migrate to the edge of the plasma membrane and fuse with the plasma membrane (PM) to release the exosomes out of the cell. This process requires the participation of cytoskeletal proteins (actin and microtubules), related molecular motors (kinesin and myosin), molecular switches (small GTPases), and fusion machinery (SNAREs). Rab proteins participate in the budding and metastasis of vesicles through their interaction with the cytoskeleton and are responsible for mediating the transport of MVBs to the plasma membrane [8]. Different types of cells have different Rab protein subtypes involved in mediating MVBs toward the edge of the plasma membrane. The transfer [12–14] of SNARE protein can form a complex with SNAPs between two membranes and mediate the membrane fusion between two organelles. SNARE protein can promote the fusion of MVBs with the plasma membrane [15]. Secreted extracellular exosomes can be taken up by the recipient cells by a variety of pathways. First, exosomes bind to the receptor cell surface through specific surface receptors [16, 17]; they are then internalized by receptor cells via endocytosis, endocytosis or phagocytosis, and plasma membrane fusion [18, 19], thereby releasing the contents into the cytoplasm of the recipient cells and exerting their biological functions.

## 3. Exosome Contents and Functions

Exosomes secreted by living cells contain a variety of biologically active substances, and 9769 proteins, 2838 miRNAs, and 1116 lipids have been discovered (ExoCarta database). The contents of exosomes are highly variable and depend on the origin of the cells but differ from them. More importantly, exosomes can transport these biologically active substances to adjacent or distal cells and exert their corresponding biological functions, thereby altering the biological behavior of the recipient cells.

### 3.1. Protein

Since exosomes originate in the endosome, some endosomal-associated proteins are ubiquitously present in exosomes, including membrane transport and fusion-associated proteins (Rab-GTPase, annexin, and heat shock proteins (HSPs), including Hsp60, Hsp70, and Hsp90), four-transmembrane cross-linking proteins (tetraspanins) (including CD9, CD63, CD81, and CD82) and MVB-related proteins (Alix and TSG101) (widely used for the identification of exosomes [4, 20]). Sourced exosomes also carry specific proteins associated with tumor cells. Exosomes released by glioma cells (GBMs) are rich in cytokines, such as VEGF-A, semaphorin-3A, and TGF-*β* [21], as well as those released by GBM and lung cancer cells. The presence of EGFRvIII is found in cell-derived exosomes [22]. HIF1*α* is highly expressed in nasopharyngeal carcinoma-derived exosomes (associated with tumor cell proliferation, migration, and angiogenesis) and promotes receptor cell migration [23]. MT1 MMPs are contained in exosomes derived from fibrosarcoma and melanoma cells and activate MMP2 in the recipient cells, thereby altering the extracellular matrix of the recipient cells [24]. Studies have also revealed that ovarian cancer cells are derived from exosomes. Exosomes contain FasL and TRAIL that can induce dendritic cells (DCs) and peripheral blood mononuclear cells (PBMCs) to undergo apoptosis, which causes immunosuppression and promotes tumor progression [25]. Interestingly, exosomes also contain cell signaling pathway-associated proteins, such as Wnt proteins [26, 27] and Notch ligand DLL4 [28], and proteins that mediate cell-cell communication, such as interleukins [29]. These proteins play an important role in tumor development, maintenance, and resistance.

### 3.2. Nucleic Acid

With the application of next-generation technologies such as high-throughput sequencing in biology, a large amount of genetic material is found in exosomes. Studies have found that exosomes are rich in small noncoding RNAs (including miRNAs, snRNAs, and snoRNAs) [30] and also contain specific mRNAs, but contain only extremely low levels or undetectable 18s and 28s RNA [6]. Mitochondrial DNA and small fragments of DNA sequences have even been found in EVs [31, 32]. miRNAs are small noncoding RNAs of 20 to 25 nt in length that are involved in the posttranscriptional regulation of genes by targeting mRNA sequences. The study found that the process of miRNA sorting into exosomes is not random. The type and expression level of miRNAs in exosomes are related to their origin but are different. Guduric-Fuchs et al. [33] analyzed the expression levels of miRNAs in various cell lines and their released exosomes and found that some miRNAs (e.g., miR-150, miR-142-3p, and miR-451) were prioritised and selected by exosomes; Ohshima et al. [34] also found that the let-7 miRNA family was abundantly present in gastric cancer cell line AZ-P7a-derived exosomes, but not in the exosomes of other tumor cells. Tumor cell-derived exosomes were found to contain tumor cell-specific miRNAs. The exosomes of metastatic breast cancer cells are rich in miR-200s and can enhance the metastatic ability of nonmetastatic tumor cells [35]; GBM-derived EVs are rich in many tumorigenic miRNAs, including miR-21, miR-23a, miR-30a, miR-221, and miR-451. By transporting these miRNAs, GBM-EVs can alter the phenotype of nonmalignant cells in their microenvironment and promote tumor progression. However, tumor suppressor miRNA was also found in tumor cell exosomes, such as miR-34, which promotes apoptosis and senescence, and was found in exosomes released from breast, prostate, brain, and bladder cancer cells [36, 37].

Long noncoding RNA (LncRNA) is a type of RNA molecule with a transcript of more than 200 nt in length. They do not encode proteins but participate in chromosome modification, gene transcription, and mRNA translation in the form of RNA and the regulation of protein function [38]. Gezer et al. [39] identified six LncRNAs MALAT1, HOTAIR, lincRNA-p21, GAS5, TUG1, and CCND1-ncRNA in HeLa and MCF-7 cells and their secreted exosomes. It was found that there were differences in the expression levels of these six LncRNAs in the nucleus of their exosomes. Kogure et al. [40] also found a new ultraconservative LncRNA (ucRNA) TUC339 in the exosomes of hepatoma cells. In addition, circular RNA (circRNA) with miRNA sponge function was also found in exosomes of tumor cells, and the expression level in exosomes was much higher than that in the source cells [41]. The mRNA carried by exosomes is far less abundant than the miRNAs carried by the source cells, and its expression level in exosomes is also different from that in the source cells. Studies have found that mRNA carried by exosomes is translated into proteins in receptor cells and exerts its function [42].

### 3.3. Lipids

Compared with proteins and nucleic acids, the lipid composition of exosomes is less studied. In general, the lipid composition of EVs (including exosomes) shares common features with the cells of origin. However, some studies have also found that different types of extracellular vesicles contain some specific lipids. Exosomes are rich in sphingomyelin, phosphatidylserine (PS), cholesterol, and saturated fatty acids [43]. It has also been found that ganglioside GM3 and ceramides and their derivatives are also enriched in exosomes [44]. Among them, sphingomyelin, cholesterol, and GM3 can enhance the rigidity and stability of the exosomal membrane [45]. However, LBPA present in ILVs was not enriched in exosomes. Huarte [38] found that the content of diacylglycerol and sphingomyelin in MSCs derived from bone marrow was higher, while the content of ceramide was relatively low. The lipid and lipid-metabolizing enzymes of exosomes were found to participate in the occurrence and release of exosomes and the sorting of the contents. Trajkovic et al. [46] found that inhibition of the activity of neutral sphingomyelinase (nSMase) by inhibitors or siRNAs reduced the release of exosomes. Phuyal et al. [47] found that increasing the level of E-thermic lipids in prostate cancer cells not only promotes secretion of exosomes but also changes the lipid and protein components of exosomes. The contents of the exosomes carried by the exosomes differed from those of the cells, and they had originated in both species and expression levels, indicating that the exosomal contents were selectively sorted into the ILVs.

## 4. Cancer Stem Cells and Plasticity

### 4.1. Cancer Stem Cell Model

A large number of experimental studies have shown that tumor tissue is composed of a variety of heterogeneous tumor cells, and there is a small group of tumor cells with stem cell characteristics, namely, CSCs or cancer-initiating cells (CICs). Because of their self-renewal capacity, multidifferentiation potential, and tolerance to radiochemotherapy, cancer stem cells are considered to be the root cause of tumor growth, development, and recurrence. The earliest evidence for the existence of cancer stem cells stems from the study of malignant leukemia. Shimada et al. [48] studied primary lymphoma cells in mice and found that a small group of cells in the transplanted lymphoma cells had stem cell properties and was able to form colony subpopulations on the spleen of the recipient mice. Subsequently, Hamburger and Salmon [49] and Bonnet and Dick [50] also confirmed that a small fraction of cancer cells in mouse or human primary tumor tissues had indefinite proliferative capacity by means of soft agar cloning. The results confirmed that they have a self-renewal ability. The presence of competent malignant leukemia cells provides direct evidence for the cancer stem cell hypothesis. Subsequently, the researchers successfully isolated the CD34+, CD38− [51], and CD90− [52] phenotypes of leukemia cells from acute myelocytic leukemia (AML) tissues by immunofluorescence and flow cytometry. Only this subpopulation of cells was found to form AML in NOD/SCID mice. Al-Hajj et al. [53] used the same method to isolate CD44^+^ CD24^−^/low lineage− cancer cells from breast cancer tissues and found that this subpopulation of cells could sustain tumor formation in NOD/SCID mice. And there is a strong tumorigenicity. The remaining tumor cells failed to form tumors even when they were injected several times with the number of cells. In addition, the researchers succeeded in isolating and identifying cancer stem cells from human glioma tissue by using surface molecules (CD133, CD44) [54, 55], side population sorting, and tumor globulogenesis analysis [56]. Subsequently, the presence of cancer stem cells was also found in a series of solid tumors [57, 58]. The cancer stem cell hypothesis states that cancer stem cells are the only small group of cells that have a starting tumor and promote tumor growth. Over the past several decades, cancer stem cells have been discovered in hematologic and solid tumors. Cancer stem cells have a self-renewal ability and multidirectional differentiation potential and are resistant to radiochemotherapy. The dry nature of cancer stem cells is considered to be the root cause of traditional tumor treatment failure and tumor recurrence. Therefore, targeting cancer stem cells may be a new and effective method for radical tumors.

### 4.2. Plasticity of Cancer Stem Cells

Cancer stem cells have a self-renewal ability and multidirectional differentiation potential, and they differentiate to produce a variety of tumor cell subpopulations with limited proliferative capacity, which can distinguish or identify cancer stem cells. However, recent studies have found that the differentiation of cancer stem cells into nonstem tumor cells (non-CSCs) may not always be a one-way, irreversible process as previously recognized; instead, the cancer stem cell phenotype has plasticity, and some differentiated tumor cells can be transformed from a nonstem cell state to a stem cell state and noncancer stem cell subpopulation cells can also be transformed into cancer stem cells [59, 60].

Vermeulen et al. [61] found that non-tumor-initiating cells isolated from colon cancers reexpress CSC markers after coculture with fibroblasts and restored tumorigenicity, suggesting that the stemness of tumor cells is not immutable and can be regulated. Roesch et al. [62] found that even a single JARID1B-melanoma cell can produce a variety of heterogeneous progeny cells including JARID1B+ cells (having cancer stem cell characteristics). In studies of gliomas, it was also found that temozolomide-treated nondry GBM cells can be dedifferentiated to obtain a stem cell phenotype and potency [63]. In other types of tumors, it has also been demonstrated that differentiated tumor-reducing tumor cells can regain a stem cell-like phenotype by various means [64, 65]. The above findings suggest that cancer stem cells may not be a stationary cell population but are dynamically changing populations. On the one hand, cancer stem cells constantly undergo self-renewal and differentiation to produce nondrying tumor cells. On the other hand, differentiated tumor cells are continuously dedifferentiated, and stem cells are obtained as stem cells to maintain the dynamic balance and tumor growth of stem cells in tumors and relapse. At present, the mechanism of regulation of the dynamic balance of cancer stem cells is not clear, but studies have found that some factors can induce nondrying tumor cells into stem cells.

### 4.3. Factors Affecting Plasticity of CSC

#### 4.3.1. Cancer Stem Cell Microenvironment

Normal stem cells (NSCs) need to rely on special stem cell niches to maintain their stem cell characteristics, such as self-renewal and multilineage differentiation potential [66, 67]. Similarly, cancer stem cells also require the same special microenvironment the cancer stem cell microenvironment (CSC niche) to maintain a balance between self-renewal and differentiation [68]. The molecular cross-talk between cancer stem cells and their microenvironment plays an important role in maintaining their stem cell phenotype and function. Mesenchymal stem cells (MSCs) are one of the important components of the cancer stem cell microenvironment and can secrete a variety of cytokines, providing a favorable microenvironment for the generation of cancer stem cells. Breast cancer stem cells secrete IL-6, recruit mesenchymal stem cells, and induce their production of CXCL7 cytokines to support the cancer stem cell phenotype [69], and IL-6 can also induce differentiated tumor cells to transform into tumor stem cell phenotypes [70]. MSCs can also upregulate the expression level of miR-199a through direct contact with breast cancer cells, cause a series of abnormal expression of related microRNAs, and inhibit the expression of FOXP2, thereby improving the stem cell characteristics of tumor cells [71]. The interaction between cancer stem cells and endothelial cells is also crucial for the maintenance of the phenotype of cancer stem cells and their function. Endothelial cells in the tumor microenvironment can regulate the biological behavior of cancer stem cells through direct interaction with tumor cells or release of cytokines [72]; vascular endothelial cells can activate glioma stem cells by secreting nitric oxide (NO). The NOTCH signaling pathway promotes the self-renewal of tumor stem cells and inhibits their differentiation [73]. In addition, hepatocyte growth factors (HGF) [61] and annexin A1 [74] released by cancer-associated fibroblasts (CAFs) can restore differentiated tumor cells to stem cell phenotypes. Santisteban et al. [75] found that CD8+ T cells can promote the epithelial-mesenchymal transition (EMT) process of breast cancer cells to obtain the characteristics of cancer stem cells, including high tumorigenicity and resistance to radiotherapy and chemotherapy. In addition, hypoxic conditions in the tumor microenvironment can also induce stem cell phenotypes in nondrying tumor cells [76, 77]. The tumor stem cell microenvironment is an indispensable factor in the proliferation, differentiation, and survival of cancer stem cells. The interaction of the microenvironment facilitates the maintenance of their dynamic balance.

#### 4.3.2. EMT and Its Transcription Factors

EMT is a phenomenon in which epithelial cells transdifferentiate into mesenchymal cells and is essential for the morphogenesis of embryos during development [78, 79]. More importantly, the activation of the EMT process is closely related to the normal and maintenance of the dryness of the tumor cells. A large number of experimental studies have confirmed that EMT can induce the transformation of tumor cells into cancer stem cells [80, 81]. Overexpression of EMT-associated transcription factors Snail, Twist, or FOXC2 not only enables phenotypic transformation of breast cancer cells but also enhances tumor glomus formation, soft agar clonality, and tumorigenic potential and enables them to obtain CD44high/CD24low (cancerous CSCs and markers for mammary epithelial stem cells) antigen phenotype [82, 83]. This indicates that the EMT process plays an important role in the transition of a cancer stem cell state. Studies in tumor cells have revealed that the repressor of E-cadherin ZEB1, a key regulator of epithelial-mesenchymal transition, can promote the dedifferentiation of invasive ductal and breast lobular carcinoma by inhibiting epithelial polarity [84]. Chaffer et al. [64] also confirmed that the EMT transcription factor ZEB1 can mediate nondry basal-like breast cancer cell (CD44low) to stem cell state transition (CD44high) and also found that the EMT inducer TGF-beta can effectively promote basal nonstem cells in stem-like breast cancer cells which are transformed into stem cells. In addition, Fang et al. [85] found that another EMT transcription factor, Twist2, promotes the self-renewal capacity of breast cancer stem cells. Overexpression of Twist2 can increase the ability of breast cancer cells and mammary epithelial cells to form colonies, promote tumor growth, and increase the number of CD44high/CD24low cell subpopulations and stem cell marker expression.

In addition, some miRNAs [86] and cytokines and growth factors secreted by stromal cells in the tumor microenvironment [87] can also induce tumor cells. The EMT process transforms differentiated tumor cells into cancer stem cells.

#### 4.3.3. Reprogramming Transcription Factors

The transient expression of reprogramming transcription factors OCT3/4, SOX2, c-Myc, KLF4 or OCT4, SOX2, NANOG, and LIN28 allows differentiated cells to regain stem cell properties. These transcription factors, which play a key role in maintaining self-renewal of embryonic stem cells (ESCs), are often highly expressed in tumor tissues and can induce dedifferentiation of tumor cells to obtain a stem cell phenotype. Suvà et al. [88] identified several neurodevelopmental-related transcription factors (POU3F2, SOX2, SALL2, and OLIG2) and found that overexpression of either transcription factor induces reprogramming of differentiated GBM cells into stem cell-like GBM cells. Oshima et al. [89] found that transfection of OCT3/4, SOX2, and KLF4 transcription factors enhances the dry phenotype of colon cancer cells; Zbinden et al. [90] and Jeter et al. [91] confirmed the overexpression of the transcription factor NANOG. It can induce tumor stem cell-like phenotypes and characteristics and upregulate the expression levels of cancer stem cell markers CXCR4, IGFBP5, CD133, and AL-DH1. Downregulation of NANOG expression inhibits tumor cell self-renewal and tumorigenicity. It has also been found that the reprogramming transcription factors OCT4 and SOX2 are highly expressed in undifferentiated cancer stem cell subpopulations and play an important role in the maintenance of the stem phenotype of cancer stem cells. Overexpression of OCT4 can induce the dedifferentiation of melanoma cells, transform into stem cell-like phenotypes, acquire the ability to form tumor spheres, increase drug resistance, and increase tumorigenicity in vivo [92]; Murakami et al. [93] also found overexpression of OCT4 and SRY. It can enhance the stem phenotype of liver cancer cells, while downregulating the expression level of OCT4 will inhibit the stem cell characteristics of tumor cells [94]. In the study of glioma (GBM), it was found that overexpression of SOX2 can significantly enhance the stem cell phenotype of GBM [95], while silencing the expression of SOX2 by RNAi technology can inhibit the proliferation of GBM stem cells and deactivate them [96, 97]. In other tumors, it has also been found that SOX2 induces dedifferentiation of tumor cells and confers a stem cell-like phenotype [98, 99]. The above results indicate that reprogramming transcription factors can regulate the plasticity of cancer stem cells and play an important role in maintaining the dynamic balance of cancer stem cells.

## 5. The Role of Exosomes in Maintaining the Phenotype of Cancer Stem Cells

As mentioned above, differentiated nonstem tumor cells and cancer stem cells can be transformed into each other, maintaining the dynamic balance of cancer stem cells. The “cross-talk” between tumor cells, cancer stem cells, and their microenvironment is an important site and material basis for this dynamic homeostasis. Exosomes, as carriers of biologically active substances, mediate many types of cellular communication, and it can be speculated that exosomes may regulate cancer stem cell differentiation and tumor cell dedifferentiation by transporting stem-related-specific molecules, thereby maintaining tumor stem cell homeostasis. See Table 1 for details.

### 5.1. Exosomes Mediate Communication between Tumor Cells and Their Microenvironments

The substance exchanges and signal communication between cancer stem cells and tumor cells and stromal cells in the tumor microenvironment are crucial in the maintenance of the dynamic balance of cancer stem cells, while the release and uptake of extracellular vesicles (EVs) is an important way to mediate information exchange between tumor cells and between tumor cells and their microenvironment [100]. Recent studies have found that exosomes released by cancer-associated fibroblasts (CAFs) in the tumor microenvironment can promote the phenotypic enhancement of differentiated tumor cells [101] and can regulate the survival and proliferation of tumor cells [102]. Rodríguez et al. [103] also found that the exosomes released by stem cell-like breast cancer cells are rich in stem- and metastasis-associated mRNA and can promote the tumorigenic potential of the recipient cells.

### 5.2. Exosome-Mediated Dry Pathway

**Figure 1:**
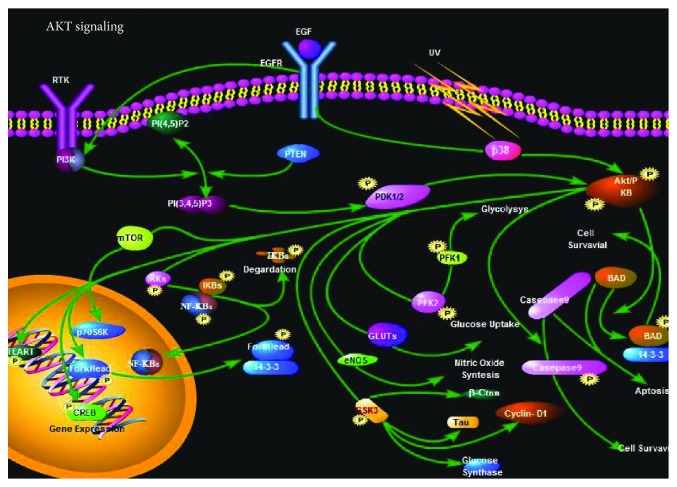
Gastric cancer cell-derived exosomes can promote tumor cell proliferation through PI3K/Akt signaling pathways.

**Figure 2:**
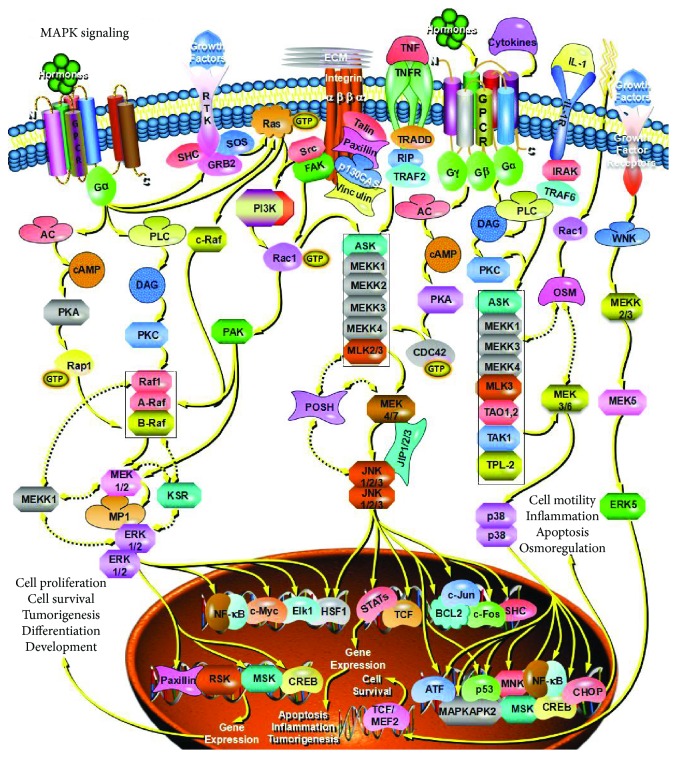
Gastric cancer cell-derived exosomes can promote tumor cell proliferation through MAPK/ERK signaling pathways.

Wnt signaling plays an important role in many biological processes such as growth, development, metabolism, and stem cell maintenance. The abnormal activation of the Wnt pathway is closely related to the development of the tumor [104] and is involved in the regulation of CSC self-renewal and differentiation [105–107]. Recent studies have found that exosomes can mediate the regulation of the Wnt pathway in recipient cells. In studies of colorectal cancer (CRC), it has also been found that exosomes of fibroblasts activate the Wnt signaling pathway of CRCs, allowing CRCs to exhibit stem cell properties, including spherocytosis and tumorigenicity, and increases the proportion of CSCs in CRCs [108]. Similarly, exosomes derived from mesenchymal stem cells (MSCs) can also promote breast cancer cell proliferation by activating the Wnt signaling pathway [109]. The exosome secreted by the collateral cells of lymphoma can transport the Wnt signaling pathway in Wnt3a-activated receptor cells, mediating the transformation between the side-group cells and nonside population cells [26]; furthermore, the study also found that gastric cancer cell-derived exosomes can promote tumor cell proliferation through PI3K/Akt (Figure 1) and MAPK/ERK signaling pathways (Figure 2) [110]; exosomes released from stromal cells can activate Notch3 signaling pathways in breast cancer cells and enhance the therapeutic tolerance of breast tumor cells. Sex [111].

### 5.3. Exosomes Induce EMT

The EMT process plays an important role in regulating the self-renewal and differentiation of CSCs, and cells can obtain stem cell phenotypes through EMT processes. Transforming growth factor beta (TGF-beta), which is capable of inducing the onset of EMT, was found in tumor cell-derived exosomes in recent studies. For example, chronic myeloid leukemia- (CML-) derived exosomes are rich in TGF-*β*1, transport TGF-*β*1 through exosomes, and promote leukemic cell proliferation, colony formation, and tumor formation in vivo [112]. The exosomes released by colon cancer-initiating cells transport cld7 into low metastatic cells, inducing their EMT process [113].

### 5.4. Transport Reprogramming Transcription Factor

Aberrant expression of reprogramming transcription factors can induce the conversion of non-CSCs to CSCs, and exosomes can regulate the dynamic balance of cancer stem cells by transporting these transcription factors or by regulating the expression levels of transcription factors in the recipient cells. For example, exosomes secreted by preadipocytes promote early breast cancer formation and tumor growth in vivo by transporting the transcription factors SOX2 and SOX9 [114]. In addition, the miRNAs contained in exosomes also play an important role in the regulation of tumor cell proliferation, self-renewal, and tumorigenicity. High expression of miR-222 in melanoma cell-derived exosomes can increase the malignant phenotype of melanoma cells [115]; gastric cancer cells can selectively encapsulate Let-7 miRNAs into exosomes and release them into the tumor microenvironment, thereby promoting the malignant phenotype and tumor growth of gastric cancer [34]. Other oncogenic miRNAs (oncomiRs), such as miR-21 [116] and miR-34a [117], have also been found to be abundant in tumor cell-derived exosomes.

## 6. Targeting Exosomes and Tumor Therapy

In conclusion, exosome-mediated cell communication plays an important role in tumor development and tumor stem cell homeostasis. Therefore, blocking the exosome's biogenesis, release, translocation, and signaling pathway is likely to become another new tumor-targeted treatment.

### 6.1. Inhibition of Exosome Biogenesis

Recent studies have found that several key proteins involved in the development of exosomes, such as ESCRT, are involved in the formation of MVBs and ILVs [9]. Several studies have found that knockout of HRS, STAM1, and TSG101 can reduce exosome release and inhibition of these ESCRT components can alter vesicle properties and contents [118]. In addition to the production of exosomes via an ESCRT-dependent pathway, sphingolipid ceramide also mediates the production of exosomes and a hydrochloride hydrate (GW4869) can induce the inactivation of the acid sphingomyelinase (aSMase). Treatment of cells attenuates endosomal body sorting and production [119]. In addition, tetraspanin can mediate the production of MVBs, and expression of Tspan8 in rat pancreatic cancer cells can alter the mRNA content and protein composition of exosomes [120]. If it interferes or inhibits the expression of four-transmembrane cross-linking molecules, it is possible to inhibit the exocrine biogenesis of pancreatic cancer cells.

### 6.2. Inhibition of Exosome Release

The Rab27 family is a class of small GTPase proteins that play an important regulatory role in the release of exosomes. The inhibition of Rab27a expression by interfering RNA technology can reduce the release of exosomes from tumor cells and inhibit the growth of tumors and the formation of metastatic clones [121]. Other Rab proteins, such as Rab11 and Rab35, can also weaken the release of exosomes by inhibiting the binding of MVBs to the plasma membrane [12, 13]. In addition, some lipids have also been shown to be involved in regulating the release of exosomes. Studies have found that downregulation of diacylglycerol kinase alpha inhibits the release of exosomes containing the Fas ligand [122]. The release of most extracellular exosomes correlates with the concentration of intracellular Ca^2+^, and increasing the concentration of intracellular Ca^2+^ can stimulate the release of exosomes, while inhibiting Na^+^/Ca^2+^ exchange channels. The agent dimethyl amiloride (DMA) attenuated the release of exosomes caused by elevated Ca^2+^ concentration [123].

### 6.3. Inhibition of Exosome Uptake

Cells can take exosomes in the extracellular environment into the cell through multiple pathways, including endocytotic receptor-mediated endocytosis and direct fusion with the plasma membrane, although the mechanism of cell internalization of exosomes is also not entirely clear. Some studies have found that uptake of tumor-derived EVs seems to be related to phosphatidylserine on its surface [124, 125]. Other studies have found that EVs in GBM cells can be mediated through heparin proteoglycans (HSPGs) present on the recipient cells. Treatment of cells with heparin can interfere with the binding of EVs to the recipient cells, thereby inhibiting the phenotypic changes induced by EVs [126, 127]. In addition, by downregulating some of the proteins involved in endocytosis, such as dynein 2 [19], the uptake of exosomes by receptor cells is also inhibited.

In summary, the inhibition of the occurrence, release, and uptake of exosomes can provide new potential targets for the treatment of tumors. However, the problem is how to specifically interfere with these pathways of tumor cells without affecting the occurrence, release, and uptake of normal extracellular secretions. Therefore, future studies should look for strategies and methods that specifically inhibit the occurrence, release, and uptake of exosomes by tumor cells to enhance their specificity and targeting of tumor cells. See Table 2 for details.

## 7. Summary and Outlook

**Figure 3:**
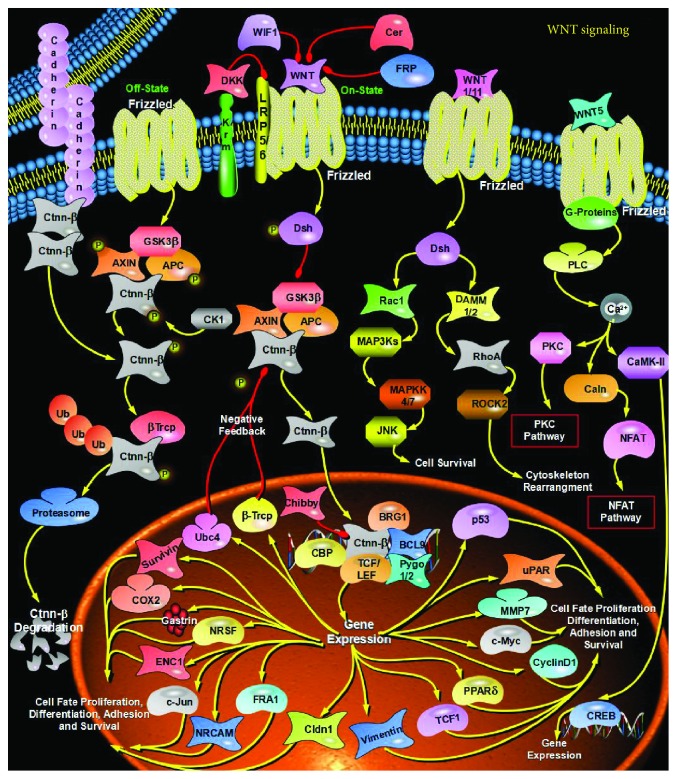
Exosomes can regenerate stem cell phenotypes and convert them to CSCs by regulating the Wnt pathway.

**Figure 4:**
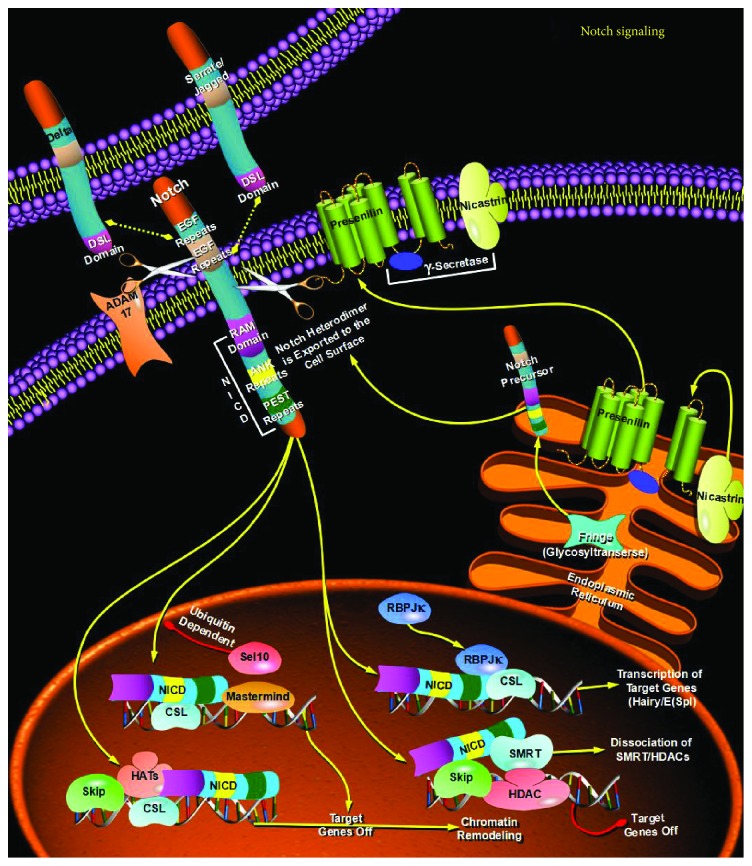
Exosomes can regenerate stem cell phenotypes and convert them to CSCs by regulating the Notch pathway.

**Figure 5:**
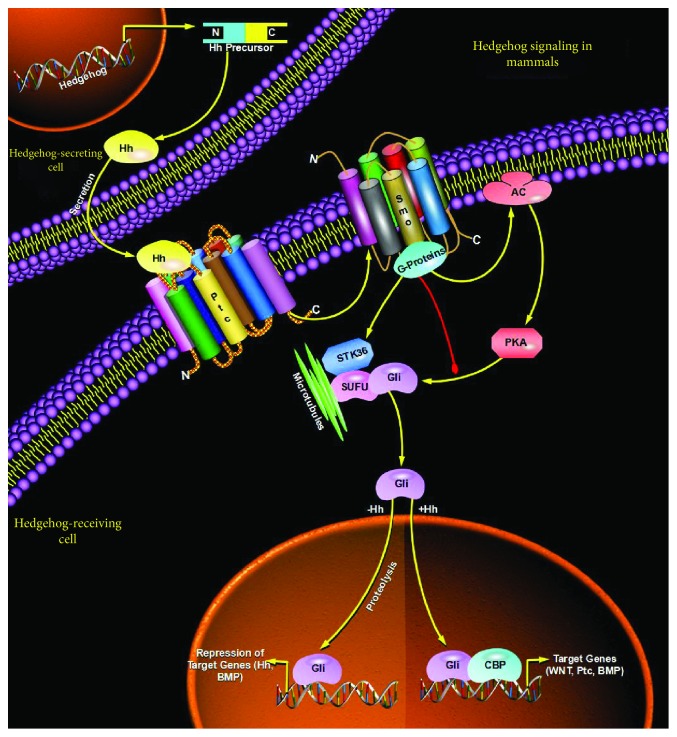
Exosomes can regenerate stem cell phenotypes and convert them to CSCs by regulating the Hedgehog pathway.

Cancer stem cells (CSCs) are a group of tumor cells with stem cell-like properties that can drive the growth and recurrence of tumors and are resistant to many current treatments. The proposed CSC hypothesis will have an important impact on clinical treatment strategies. However, more and more studies have shown that CSCs are a group of differentiated and dedifferentiated dynamic equilibrium cells, which can explain why a single anticancer or anti-CSC drug cannot kill all tumor cells or CSCs. Exosomes are nanoscale vesicles that are secreted by living cells to the outside and can regulate the gene expression and signaling pathways of receptor cells by transporting their contents, thereby mediating cell-cell communication and participating in various processes of non-CSCs and CSCs. Non-CSCs and CSCs can be interconverted, maintaining their dynamic balance. Exosomes can regenerate stem cell phenotypes and convert them to CSCs by mediating receptor cells undergoing EMT or by regulating stem-related signaling pathways (e.g., Wnt pathway (Figure 3), Notch pathway (Figure 4), and Hedgehog pathway (Figure 5)) and other pathways. They participate in the mutual transformation between non-CSCs and CSCs and maintain their homeostasis. Therefore, we speculate that exosomes may act as regulators of the homeostasis between non-CSCs and CSCs. On the one hand, exosomes derived from CSCs can transfer dry molecules to non-CSCs to give them a dry phenotype; on the other hand, stromal cells in tumor cells or tumor microenvironment can also increase the formation of CSCs and promote tumor progression through exosomes. Exosomes may also serve as an information carrier to maintain the dynamic balance between non-CSCs and CSCs. On the one hand, exosomes derived from CSCs can transfer dry molecules to non-CSCs, giving them a stem cell phenotype; on the other hand, tumor cells or other cells in the tumor microenvironment can also be released or taken into account. The secretory body promotes the formation of CSCs and tumor progression.

## 8. Conclusions

In conclusion, exosomes serve as information carriers and play an integral role in maintaining homeostasis between non-CSCs and CSCs. Targeting the inhibition of exosome biosynthesis or disrupting the formation, release, and uptake of exosomes and blocking the dynamic transformation and homeostasis between non-CSCs and CSCs thereby eliminate cancer stem cells and eradicate tumors.

## Figures and Tables

**Table 1:** Role of exosomes in maintaining a stem cell phenotype of tumor cells.

Donor cells	Related molecules contained	Receptor cells	Action route	Features	References
Lymphoma cells	Wnt3a	Lymphoma cells	Wnt pathway	Enhance the cloning ability of side population cells and mediate conversion between side population cells and nonside population cells	[26]
CAFs	—	CRCs	Wnt pathway	Increased CRCs into balls and tumorigenicity, increased proportion of CSCs	[108]
MSCs	#VALUE!	Breast cancer cells	Wnt pathway	Promote breast cancer cell proliferation and migration	[109]
CMLs	TGF-*β*	CMLs	Wnt pathway	Promote tumor cell proliferation, colony formation, and tumor formation in vivo	[112]
CoCa	cld7	CoCa	EMT	Induce EMT and enhance metastasis and invasion of low metastatic tumor cells	[113]
Preadipocytes	SOX2/SOX9	Breast cancer cells	—	Promote early breast cancer formation and tumor growth in vivo	[114]
CAFs	miR-21 miR-378e miR-143	Breast cancer cells	—	Enhance receptor cell dryness and EMT phenotype	[101]
Melanoma cells	miR-222	Melanoma cells	PI3K/AKT pathway	Increase the malignant phenotype of melanoma cells	[115]
Esophageal cancer cells	miR-21	Esophageal cancer cells	PDCD4	Promote receptor cell migration and invasion	[116]
Stem cell-like breast cancer cells	mRNA	Breast cancer cells	—	Promote breast cancer development and metastasis	[103]

**Table 2:** Targeted exocrine and possible pathway of tumor therapy.

Action pathway	Action target	Function	References
Biogenesis	ESCRT components	Reduce the release of exosomes and change their content components	[118]
	aSMase	Decrease the sorting and production of exosomes	[119]
	Tetraspanin	Reduce the production of MVBs and change the mRNA content and protein composition of exosomes	[120]
	Rab protein	Inhibit the binding of MVBs to the plasma membrane	[12, 13, 121]

Release process	Diacylglycerol kinase alpha	Inhibition of the release of exosomes containing the Fas ligand	[122]
	Na^+^/Ca^2+^ channel	Reduce the release of exosomes	[123]
	Phosphatidylserine	Interfere with the binding of EVs to receptor cells	[124–127]

Ingestion process	Dynein	Inhibition of exosomal uptake by receptor cells	[19]

## Abbreviations

CSCs: Cancer stem cells
MVBs: Multivesicular bodies
ILVs: Luminal vesicles
ESCRT: Endoprotein sorting and transferring device
PM: Plasma membrane
HSPs: Heat shock proteins
PBMCs: Peripheral blood mononuclear cells
DCs: Dendritic cells
CICs: Cancer-initiating cells
NSCs: Normal stem cells
AML: Acute myelocytic leukemia
MSCs: Mesenchymal stem cells
CAFs: Cancer-associated fibroblasts
HGF: Hepatocyte growth factors
EMT: Epithelial-mesenchymal transition
EVs: Extracellular vesicles
CRC: Colorectal cancer
TGF-beta: Transforming growth factor beta
aSMase: Acid sphingomyelinase
HSPGs: Heparin proteoglycans.
